# Feasibility of Sensor Technology for Balance Assessment in Home Rehabilitation Settings

**DOI:** 10.3390/s21134438

**Published:** 2021-06-28

**Authors:** Daniel Kelly, Karla Muñoz Esquivel, James Gillespie, Joan Condell, Richard Davies, Shvan Karim, Elina Nevala, Antti Alamäki, Juha Jalovaara, John Barton, Salvatore Tedesco, Anna Nordström

**Affiliations:** 1 Faculty of Computing, Engineering, and the Built Environment, Ulster University, Londonderry BT48 7JL, UK; kc.munoz-esquivel@ulster.ac.uk (K.M.E.); gillespie-j10@ulster.ac.uk (J.G.); j.condell@ulster.ac.uk (J.C.); rj.davies@ulster.ac.uk (R.D.); Haji_Karim-S@ulster.ac.uk (S.K.); 2 Department of Physiotherapy, Karelia University of Applied Sciences, Tikkarinne 9, FI-80200 Joensuu, Finland; Elina.Nevala@karelia.fi (E.N.); Antti.Alamaki@karelia.fi (A.A.); Juha.Jalovaara@karelia.fi (J.J.); 3 Wireless Sensors Network Group, Tyndall National Institute, University College Cork, T12 R5CP Cork, Ireland; john.barton@tyndall.ie (J.B.); salvatore.tedesco@tyndall.ie (S.T.); 4 Department of Public Health and Clinical Medicine, Umeå University, SE-901 87 Umeå, Sweden; anna.h.nordstrom@umu.se

**Keywords:** accuracy, balance, clinical diagnosis, rehabilitation, remote sensing, sensor systems, wearable sensors

## Abstract

The increased use of sensor technology has been crucial in releasing the potential for remote rehabilitation. However, it is vital that human factors, that have potential to affect real-world use, are fully considered before sensors are adopted into remote rehabilitation practice. The smart sensor devices for rehabilitation and connected health (SENDoc) project assesses the human factors associated with sensors for remote rehabilitation of elders in the Northern Periphery of Europe. This article conducts a literature review of human factors and puts forward an objective scoring system to evaluate the feasibility of balance assessment technology for adaption into remote rehabilitation settings. The main factors that must be considered are: Deployment constraints, usability, comfort and accuracy. This article shows that improving accuracy, reliability and validity is the main goal of research focusing on developing novel balance assessment technology. However, other aspects of usability related to human factors such as practicality, comfort and ease of use need further consideration by researchers to help advance the technology to a state where it can be applied in remote rehabilitation settings.

## 1. Introduction

Health care services are facing demands relating to an increased number of elderly people becoming physically inactive [1]. They are also having to deal with frailty, diabetes, neurodegenerative or cardiovascular diseases, and injuries linked to falling that result in cognitive, physical and psychological consequences, such as dementia, lack of independence and isolation. Remote rehabilitation presents a potential solution to deal with the increased health care service demands in relation to a growing elderly population [2]. Remote rehabilitation has the potential to enhance the quality of service, decrease costs and reduce the demand for resources such as nurses, health practitioners, specialists, rooms and beds. Patients who live in remote and scarcely populated areas can have widening access to services while avoiding unnecessary travel [3]. The success of remote rehabilitation depends heavily on the practicality and usability of the technology—in particular wearable sensor systems [4]—and consequently the quality of the service that can be provided using the sensors. The quality of service is highly interconnected with the capabilities of the technology and the clinical effectiveness of the algorithms, but also relies on human factors such as the health practitioner’s knowledge on how to operate these technologies and on how to interpret the information appropriately. Technologies used for remote rehabilitation provide easier access to vital health care services such as physiotherapy. The economic case for the use of these technologies in averting falls and fractures in the elderly population is clear. For example: In 2012/2013, the average cost of treating a hip fracture in the first year was about £14,264 in the UK and the cost in Western Europe was about 14,429 euro [5].

Focusing on monitoring the physical capacity of elderly people, the smart sensor devices for rehabilitation and connected health (SENDoc) project aims to evaluate the use of wireless sensor technologies in remote rehabilitation settings. Currently, there are many off-the-shelf technologies available, while other research-led prototypes are under development. It is important to consider that many of the core sensors used as the basis to create these technologies have been developed within the last 15 years and thus provide a sound technological underpinning. What these sensors measure and where they are located depend on the actual health parameters being monitored and analysed [6,7].

One of the key parameters which can be measured using sensor technology is balance. In order to perform all activities of daily living, a person must possess good balance control while at rest or moving. Balance refers to a person’s ability to keep their centre of mass in the base of support and requires coordination of the sensory, neural and musculoskeletal systems. These systems have been shown to deteriorate as people age and issues relating to poor balance, such as reduced safe mobility and increased falls risk, are more prevalent as age increases [8].

### 1.1. Clinical Approaches to Assess Balance

While reviewing potential technologies that support remote assessment of balance is the core aim of this paper, it is important to first establish context and consider how assessment of balance is currently performed. Balance assessment is most frequently performed in clinical settings in order to analyse specific skills relating to balance control. Balance is assessed using standardised or non-standardised assessments. Standardised assessments fall into 1 of 3 categories: (1) Self-reported or clinician rated scales; (2) single-task performance measures to assess one or two important aspects of postural control; and (3) multiple task batteries. Self-reported scales rely on patients’ self-recall over a long period of time, which can be considered a weakness, while batteries require more time and supplementary administrative personnel and equipment. In terms of usability, single-task performance is more straightforward with minimal equipment needed and nominal time to complete. However, single task performance can be subjective as its administration can vary among settings, making it difficult to compare results. The Berg Balance Scale, using a battery of 14 different tasks, is commonly used as the gold standard in measuring balance and assessing falls risk [9,10].

Despite the availability of standardised assessments, research has shown that non-standardised assessments, such as observing movements and perturbations, are more commonly used by physiotherapists in real-world clinical settings. Sibley et al. (2013) found that 15.4% of physiotherapists used a standardised measure, 79.1% used non-standardised approaches and 5.5% used both approaches [11]. In addition, the research also found that, out of 357 responses from practising physiotherapists, only one used technology to aid in the assessment of balance. The authors concluded that cost, difficulties with use of the equipment and interpretation of the data are the main factors in explaining why technology solutions have such limited usage in clinical settings. It is clear that in order for clinicians to adapt assessment techniques into their day to day practice, regardless of whether the technique is dependent on technology or not, the technique must be simple, cost-effective and easy to administer. Remote assessment of balance using sensors presents a potential solution for a simple, cost-effective and easy to administer balance assessment technique if appropriate sensor devices and measurement techniques can be utilised.

### 1.2. Analysis of the State of the Art

In the literature, within the last few years, there have been a number of reviews published focusing on balance assessment technology.

Gordt et al. [12] conducted a review related to the use of training, augmented by wearable sensors, for enhancing gait, functional performance and balance. Most of the studies reviewed consider older adults both healthy and those with a condition (frailty, Parkinson etc). Diaz et al. [13] provides a review of the progress in the field of wearable technology in balance, Range of Motion (RoM) and gait. Contributions of this article include introducing a taxonomy for rehabilitation assessment, discussing various aspects of wearable sensor technologies (energy consumption, obtrusiveness, cost etc.) and discussing parameters used to define ROM, balance and gait. Leirós-Rodríguez et al. [14] reviewed articles that explore the use of accelerometers in assessing elderly balance. In particular, the review focused on early identification of fall risk, exploring gait and static balance and balance level classification. The authors state that accelerometers are more efficient in assessing balance than force platform as they are more affordable, easier to implement and quicker in providing measurements.

In terms of specific pathologies, Parkinson’s Disease (PD) is a condition that may greatly benefit from innovative sensor monitoring technologies related to balance. Hubble et al. [15] review sensor technologies to assess standing balance and walking stability in patients with PD. The review indicates that gait/balance assessment technology enables more accurate stratification of patients based on fall risk when compared to clinical tests such as Timed Up and Go (TUG) and Berg Balance Scale (BBS) etc. Porciuncula et al. [16] conducted a review of general wearable sensors systems, not exclusively balance assessment, in clinical applications and settings such as orthopaedic and neurologic rehabilitation.

Ghislieri et al. [17] conducted a detailed review of 47 research articles focused on sensors used for measuring/assessing standing balance. Characteristics of the sensor systems such as sensor type/placement, parameters and validation methods were reviewed. A conclusion made by the authors was that sensor technology could enable easy balance assessment in clinics and remote settings. However, the authors did not go as far as to assess practicality and usability issues that could affect the adaption of the technology in clinics or remote based settings.

Previous work has already shown that in order for the monitoring technology to be adopted by older adults, the technology must be easy to use and not impair mobility and independence [18]. Usability challenges must therefore be addressed in order to implement a technology that is practical, unobtrusive, well-received by older adults, and ultimately achieves health benefits. However, despite the growing number of articles supporting the use of sensor technology for balance assessment, there are still significant gaps in our understanding of the technology. In particular, existing literature reviews in the area do not consider human factors associated with potential real-world use of balance assessment technology.

The number of commercially available sensor systems, including those capable of assessing balance, has increased significantly over the last decade. As the number of available sensors types has increased, it has become increasingly more difficult for clinicians to identify appropriate sensor systems for specific use cases including balance assessment. While considering sensors and technologies, clinicians and patients must have objective methodologies available to evaluate different balance assessment technologies available. The research objective is therefore to define a methodology that can be used to objectively evaluate different sensor systems for their potential to be adapted in remote rehabilitation settings.

## 2. Methodology

As part of the process of developing an objective assessment tool, a literature review of the state of the art was conducted on balance assessment technology. When considering real-world settings, remote rehabilitation settings are of particular relevance for balance assessment sensor technology. The review therefore considered potential barriers to adapting balance assessment technology in remote rehabilitation. Proving the real-world effectiveness of sensor systems has been a notable challenge that has deferred transferring their use from research to practice [7]. Therefore, the review focused on the current state of the art and attempts to improve understanding of balance assessment technology. Following the literature review data collection phase of this work, we will set out a methodology to construct an objective assessment rubric using data collected in the literature review.

### 2.1. Literature Review

The literature review used the five key phases for research synthesis, which are namely: (1) Identifying the research question, (2) identifying relevant studies, (3) study selection, (4) gathering data, and (5) collating, summarising, and reporting the results.

**Figure 1:**
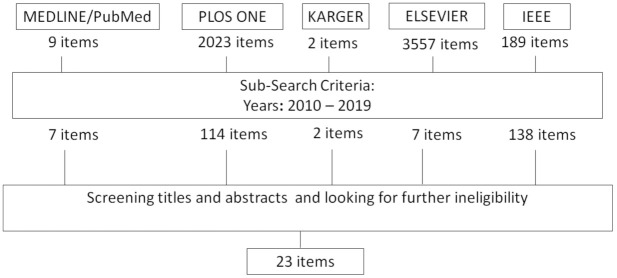
Results of Search and Screening process.

In order to identify relevant research articles for the review, a search was conducted to discover articles relating to balance assessment technology for elders. The search was carried out on well-established research databases, such as MEDLINE/Pubmed, PLOS ONE, KARGER, Elsevier and IEEE. Only publications in English were considered. The publication period investigated was 2010 to June 2019. The initial search criterion was based on the following search terms: (balance) AND (“assessment” OR “test”) AND (“older adults” OR “elderly” OR “ageing”) AND (“IMU” OR “accelerometer” OR “camera” OR “force plate” OR “wearable sensor”). Only studies specifically utilizing sensor technology such as wearable sensors, force plates and camera-based systems to assess balance in older adults were included. Studies focusing on balance interventions were not included. A second review stage involved screening titles, abstracts and publications, and those which did not meet the aforementioned criteria were excluded. Studies were only included if the sensor deployment and sensor setup procedure were sufficiently described such that characteristics of deployment and human factors could be commented on. Finally, the full texts of the remaining publications were assessed and those that were ineligible-for not covering the set criteria -were excluded. After the review stages were completed, 23 studies were finally included in this literature review. The review selection stage was conducted by one author. Figure 1 provides an overview of the paper selection phase of the review process.

Using the 23 studies, barriers to adaption were independently identified by three researchers. Identified barriers were then collated and synthesised and broad themes related to barriers to adaption were established.

### 2.2. Development of an Objective Assessment Tool

Using the themes identified in the Literature review process, the aim is to develop a scoring rubric composed of separate scoring components to objectively assess the feasibility of a particular technology being deployed in real-world remote rehabilitation settings. Based on results of the literature review, a set of independent scoring components will first be defined. For each scoring component, a clear set of criteria used to assess that component with be designed.

Each of the scoring components will be assigned a weighting factor. The weighting factors will be defined by three authors with real-world professional rehabilitation experience and who were directly involved in the literature review process described in the previous section. Each author will independently assign a weighting to each of the components. Final weights will be assigned by calculating the median of the weights assigned by the 3 independent raters.

Each criterion will be graded using a Likert Scale. After scoring, all criteria within each component should first be averaged. If a criterion cannot be assessed, due to unavailable information, that score should be marked as null and the average calculation should only consider available scores. After scores are calculated for all components, the overall score should be calculated by multiplying each score by its weighting factor and summing the weighted scores.

## 3. Results

### 3.1. Literature Review Results

Table 1, presented below, provides a summary of the key information extracted from the reviewed work during the gathering and collation phases of the review. The table details the author of each study, what the research focuses on, and a description of the study cohort. Information on the sensors or technology used for the balance assessment and how this is deployed is provided: Whether (and if so, where) it is worn on the participant or the technology is installed in the environment. The findings of the study are presented in the penultimate column. The final column discusses the human factors of the technology that the researchers in this article have inferred based on a combination of information available in the research articles and by utilising their expert knowledge of the sensors systems which have been deployed.

In order to gain further insight and understand the synergies between the state-of-the-art research in balance assessment technologies, six reoccurring key themes were identified underpinning each of the 23 research works. These were namely (1) human factors determining real-world use; (2) balance assessment methodology; (3) research objectives; (4) sensor placement; (5) analysis techniques; and (6) methods of evaluation. Detailed below is information extracted under each of these six themes.

#### 3.1.1. Human Factors Determining Real-World Use

From reviewing the 23 selected works, results show that the majority of research focuses only on one aspect of the usability of the sensors, which is their accuracy. The reviewed research does not consider, in any detailed way, aspects of usability related to human factors such as comfort, ease of mounting, technical proficiency and other practicalities related to cost, time and space.

Unobtrusive sensing is one of the key human factors that must be considered for a sensor system to be feasible in real-world home rehabilitation settings. Wearable sensors were the most frequently used technology in the reviewed works. In order for wearable sensors to be usable and practical for the intended demographic, they need to be unobtrusive, the number of sensors used needs to be minimized and the placement of the sensors on the body must allow for easy donning and doffing and be comfortable for participants to wear over long periods. In reviewing works related to wearable sensors, we gathered information related to these key characteristics such as unobtrusiveness, number of sensors, location of sensors and potential comfort and donning/diffing issues.

Five of the reviewed studies used multiple sensors placed at different locations such as two sensors at the left and right shank [20,26] and five sensors at the left shank and thigh, right shank and thigh and sacrum [29]. Discomfort and difficulty in donning and doffing are major issues relating to usability in these studies.

While many studies minimized the number of sensors to a single sensor [23,30,31,32,33,35,41,42], comfort or difficulties with donning and doffing remains a significant problem. For example, some of the sensors utilized have not been designed to be worn on the body such as Shimmer sensors and sensors in mobile phones. However researchers are employing them because they have access to the raw data and are safe to use. Elastic straps or bandages are generally used to mount sensors on the body [28,35], yet mounting sensors at very specific locations on the back is difficult to perform by participants and is generally performed by trained researchers. While easy to don and doff, waist mounted straps or bandages can be uncomfortable to wear for longer periods, and some studies can take from 24 h to one week.

Wearable sensors other than IMUs, such as wrist-worn location sensors [21], instrumented insoles [26] and Surface EMG sensors [34] were also utilized. While it is likely that a wrist-worn device may be the best option in relation to comfort and ease of donning and doffing, Kearns et al. [21] reported that 38% of older adult participants in their study disliked wearing a wristband-based device.

Similarly, insole based sensors present a potentially unobtrusive form factor for sensing gait parameters. However issues in relation to long term comfort could arise from incorrect fitting of insole to the participants shoes. Using insoles in conjunction with shoes already owned by the participant could also reduce available area for the participants feet to comfortably fit into the shoe.

Portable Surface EMG based sensors have potential to detect biological/anatomical issues, such as muscle fatigue, that could be indicative of balance issues and risk of falls. However, these technologies in their current form are still not feasible for home based rehabilitation settings. While SEMG technology has advanced in terms of miniaturisation and use or wireless technologies, donning and doffing of these sensors require placement of the electrodes at very specific anatomical sites such as M. Quadriceps Femoris [34]. This requires that an expert be present in order to set the device up.

Multiple studies are observed utilizing force plates as a comparison metric, and two of the reviewed studies [19,27] propose a force plate-based system to measure balance. Despite this, force plate-based systems are highly unlikely to be utilized in home-based rehabilitation due to installation, cost and space related issues. Camera-based motion capture systems (i.e., Vicon) were utilized in three of the reviewed studies [22,25,36]. It was observed that camera-based systems are as impractical for a home-based rehabilitation setting as the force plate systems. This is due to cost, space and installation issues. Camera-based systems are also difficult to use and require trained experts to be present to don and doff markers and operate the system software. In addition, camera-based systems could potentially be viewed as invasive from a privacy point of view.

The biggest issue in relation to feasibility of balance assessment technology being utilized in real-world home rehabilitation settings is the lack of human factors being considered by the research. While several metrics such as accuracy, specificity, reliability and test-retest reliability, are evaluated by most studies, these metrics only address one dimension of a multidimensional issue. Balance assessment technology needs to be assessed, not only in terms of accuracy, but also in terms of usability and practicality.

While there is limited quantitative evidence presented in the works related to comfort and usability, we can review participation levels in different long term study’s and make some judgement on promising sensor types for home rehabilitation. One of the most promising sensor types is a small wearable IMU sensor mounted on an elastic belt which is worn around the hip. This sensor type was successfully used in two studies to record data for up to eight days with 51 and 319 participants aged 65 and over [30,31].

#### 3.1.2. Balance Assessment Methodology

Sixteen out of the 23 research studies were related to the creation of data models and four out of 23 are related to evaluating the validity and reliability of the sensor system [19,23,25,37]. Fourteen out of the 23 studies use Inertial Measurement Unit (IMU) sensors on their own or combined with the use of other types of sensors. This corresponds to about 60.86% of all the reviewed studies. Eight out of the 23 studies (34.78% of the complete data set) focused on research conducted with Force plates, pressure sensors and insoles. Tele-surveillance and motion camera systems, and Electro Myo Stimulation (EMS) and Electrocardiograms (ECGs) were 17.39% and 13.04%, respectively. Thus, out of the reviewed studies, the most frequently researched sensors were IMU’s. These were most frequently located on the lower back or pelvis since it is close to the centre of mass of the body.

The cohort size in these studies varied from two participants in [19] to 319 participants in [31]. For most of these studies, an inclusion criteria was utilized stating that elderly healthy participants should be able to walk with and without aid for a specific period of time (e.g., 6 min in the research by Howcroft, Lemaire, and Kofman [28] or 3 min in the research by Kikkert et al. [33]), or alternatively a specific distance in meters (e.g., 20 m in the studies conducted by van Lummel et al. [30]), or be able to rise unassisted. In addition, in the majority of studies elders have to be cognitively intact [20] or perform the Mini-Mental State Examination (MMSE) and obtain a score larger than 18 or 19 out of 30 points [30,31]. When fallers were recruited, studies were asking for at least one fall in the previous 6 to 12 months. In some of these studies, the patients and their proxies were the ones reporting the time, place and circumstances of the fall. This could be seen as a weakness since they will have to recall the event with accuracy and facts. In some cases, participants did not alter normal gait owed to neurological disorders or orthopaedic injuries.

#### 3.1.3. Research Objectives

From the 23 reviewed papers, research was categorised into three key main purposes: (1) Understanding the underlying biological/anatomical functional causes creating disturbances in balance [24,32,34,36]; (2) identifying the falls or diagnosing balance issues [20,26,27,28,30,33,38,39,41,42]; and (3) identifying the factors (external or internal to a person) in order to predict when falls will happen [21,22,29,31,35].

The first category focuses on understanding if the biological/anatomical problems which create a disturbance in balance can be identified by sensors in some way. The articles in this category focus on assessing cognitive impairment linked to walking speed variation and walking trajectories [32], muscle fatigue [34], or indicators of functioning of the Central Nervous System (CNS) [36] or autonomous nervous system [24]. In order to achieve the goal of evaluating underlying biological/anatomical problems, the sensors used by the reviewed works (e.g., EMG) required complex setup and mounting at very specific body locations by an expert.

Research in the second category focused on assessing balance to identify issues in balance or a fall in order to raise an alarm or produce a notification. Trying to identify patterns of fallers from non-fallers was key for this objective. Encouragingly, works reviewed in this category commonly used light and easy to don/doff IMU sensors worn at various locations on the body to detect signals specific to fall occurrences.

The third category relates to fall prediction, where research is not only focused on internal factors to the person but can also focused on analysing the environmental, physical and socio-economic factors involved. Cameras are commonly used alongside wearable sensors to gain awareness of the environment. Many fall prediction studies focused on community dwelling environments. When multiple wearable sensors were used in addition to cameras, problems of synchronization and labelling were common, raising concerns about real-world use in rehabilitation settings. Fall prediction also requires monitoring for longer periods of times, e.g., 1 week or 24 h, or distances, e.g., 30 m. This can be challenging as time and space are the main constraints in walking tests, as can be seen in [32], and the difficulty of distinguishing the activities of daily living automatically [31,41]. Balance assessment techniques based on functional tests often require expert supervision in controlled environments. The requirement for an expert to be present clearly presents an issue in adapting the techniques for remote rehabilitation settings. A few studies did focus on remote assessment of balance without an expert [26,35], which rely on a standard test (One Leg Standing Test—OLST) or a series of standard tests (Timed-Up and Go Test—TUGT; Alternative Step Test—AST; Five Times Sit to Stand Test—FTSS) in combination with wearable sensors to assess participants. In the case of [26] research focuses on assessing the stability of the participants on different kinds of ground: Concrete, parquet, sand and gravel, and in the case of [35], the correlation of standard tests with the Berg Balance Scale (BBS) was compared (in this study *p* is equal to 0.86).

#### 3.1.4. Sensor Placement

Of the 23 studies reviewed, 14 utilized accelerometer-based technology as part of the proposed system. Eight of these studies utilized a single sensor setup where the accelerometer was mounted either around the waist with an elastic strap [30,31,33,41,42], on the back with elastic tape or bandages [23,35] or on the wrist with an elastic strap [32]. Research has also focused on identifying the most suitable location to attain an accurate measurement with the minimum number of sensors possible. This has been of particular interest since, as previously mentioned, adding wearable sensors increases the complexity in synchronization and interpretation (all combinations of sensors might need to be assessed). For example, Howcroft et al. [28] conducted experiments to assess which sensor location could be used to best identify fall risk. One IMU sensor mounted on the head performed best at identifying fall risk when participants performed a Single-Task (ST), i.e., a task without a cognitive load. The main reason behind this outcome is that the measurements attained are connected to visual input and upper body stability. After the head location, the next best location for an IMU sensor to be placed was on the pelvis or lower back as this is very near to the bodies Centre Of Mass (COM). In contrast, when participants performed a Dual Task (DT), which are tasks cognitively loaded, IMU sensors mounted on the pelvis were the most accurate when identifying fall risk. A sensor mounted on the head will clearly pose more problems for long term remote based monitoring. The pelvis location is less obtrusive, more comfortable and easier to monitor than the head location. Furthermore, smartphones equipped with accelerometers can be placed in this location easily as seen in [30]. The accuracy of the IMU sensors can be increased when combined with EMGs as can be observed in [33], reaching an accuracy of 89.7% using ten-fold cross validation in a fall detection system.

While there is a number of works which focus on identifying accurate sensor locations, few works consider the most comfortable or practical location when assessing sensor location. While it is encouraging that there is an increased focus on reducing the need for multiple sensors, the comfort and practicality of a single sensor should still be given more consideration and quantitatively evaluated.

#### 3.1.5. Analysis Techniques

Regarding the analysis techniques employed in these studies, it was found that support vector machines and neural networks were the best modelling technique for fall risk classification in [28]. For example, the best performing model was a multi-layer perceptron neural network with input parameters from pressure-sensing insoles and head, pelvis, and left shank accelerometers. This model attained 84% accuracy (F1 score = 0.600, MCC score = 0.521). One of the problem faced when analysing the data was dealing with several derived features from gait, which are highly correlated [39]. Labelling the information in order to be selected and differentiating noise from the relevant data is another challenge. Moreover, there is no consensus among researchers about the threshold that should be used in the algorithms employed. Both problems can be observed in [41]. Intra Class Correlation Coefficients (ICCs) were obtained and compared [23]. Correlations, their strength and principal component analysis were used when trying to identify the best factors to use in a model [30], or the biological functional causes of disrupted balance [24]. Logistic, Linear and Gaussian process regressions are used to create classifications models [21,32,34].

Methodologies used to train and evaluate machine learning classification models also need to be considered when assessing barriers to adaption in real-world remote rehabilitation settings. Machine learning models are known to over-fit on the training data it is provided. Inappropriate training and evaluation methodologies will therefore present an overly optimistic measurement of performance which will likely significantly reduce when presented with real-world data that it has never seen. Not using a hold out test set, optimising feature selection algorithms with data that includes test data and over reliance on cross validation are some of the common methodologies that overestimate performance [43].

For example, some fall risk prediction works use cross validation summary statistics as the only means to evaluate the model performance [20,33]. However, recent research shows that cross validation is unreliable on small data-sets (N < 1000) similar to those in the reviewed works [44]. Additionally, model and feature selection procedures are commonly ambiguous and not described in sufficient detail in a number of the reviewed works. In order to report performance metrics that will be reflective of performance in real-world conditions, it is vital that testing data is never be used to inform the model or feature selection procedure. Practices such as performing feature selection on all data (training and test data) or hyper-parameter tuning to maximise test set performance can significantly over-estimate model performance. In the relevant papers reviewed, model and feature selection procedures are frequently ambiguous and it is unclear how hyper-parameters and features were selected [33]. In order to deploy systems to uncontrolled real-world setting such as remote rehabilitation settings, the effectiveness of the system at dealing with new unseen data should be robust and evaluation procedures should be employed to reflect this.

#### 3.1.6. Methods of Evaluation

In order to assess the effectiveness of balance assessment technology, the reviewed papers commonly compare the measurements being made by the technology against an arbitrary ground truth measure. There are several different ground truth measures used in the reviewed papers. The gold standard for assessing balance is a force plate that measures postural sway through the calculation of centre of pressure (COP). COP was utilized in 5 of the reviewed studies [19,26,27,28,37].

Fall identification or prediction technologies use the number of actual falls as ground truth measures [26,31,41]. In some cases, falls were simulated as part of the study [34] and results showed that phases that are encountered in simulated falls are not found in real falls, highlighting the importance of recording data in real-life conditions. In all fall related studies, occurrence of falls is self-reported by the participant or by the participants’ family or caregivers. It is important to note that self-reported measures may be inaccurate in participants with cognitive decay.

In studies not dealing directly with falls identification or prediction, clinical balance assessments are utilized for comparison. In the reviewed papers, the most frequently used balance assessment tests for technology evaluation were BBS, TUG, STS and FTSS. Accurate timing of task performance is vital for most functional tests such as BBS and TUG. The literature shows that sensors increase the accuracy of these tests by enabling a more accurate recording of the timing of task performance. Four of the reviewed works show that instrumented tests such as TUG, Berg balance Score, STS/FTSS and Alternate Step Test (AST) are more accurate when using sensors [20,30,35,39].

While clinical balance assessments are useful for making direct comparisons with health research, the balance assessments can impose constraints upon the evaluations based on existing limitations in the design of the balance test. Due to the subjective nature of Berg Balance Scale (BBS), a minimum variation of four points in clinical assessment of BBS is needed to ensure a true change in an elder’s functional balance [45]. In spite of the quantification of movement during the TUG, using sensors might lead to a more robust method of assessing balance as seen in [20]. Some movement patterns related with fall risk might not be captured by the conventional clinical test. Short walking tests (e.g., the 10-m walking test) to assess balance are subject to bias due to their brevity while longer tests are less accepted due to space and time constraints in clinical exams [32].

It is clear that sensors can improve the accuracy of functional balance tests by removing some of the quantification errors. This is important for potential use of sensors in remote rehabilitation. By removing the need for a clinician to quantify the performance of the task, it could enable direct transfer of functional test performance from clinical settings to remote settings [26,35]. However, other issues remain, such as ensuring that patient performance of the task is correct.

Reviewing the 23 papers, and the different comparison measures used, it is evident that there is no standard measure used to evaluate balance. The measure employed is dependent on several factors including; study goals, time, space and facilities available, participants’ physical ability and researchers’ preferences. Of the 23 reviewed papers, no paper specifically evaluated the technology in terms of its usability or practicality.

### 3.2. Objective Assessment Components

Based on results of the literature review process, it is clear that both system accuracy and human factors must be considered when assessing a particular technology. While techniques to assess system accuracy, such as comparison with BBS, are well documented, objective assessment of human factors is less defined. Analysis of results from the literature review shows that the current state of the art in balance assessment technology needs to address a number of critical components required for adaption in remote rehabilitation. Based on the literature review, and analysis of the six key themes, four scoring components that should be assessed when evaluating the feasibility of a sensor system for remote rehabilitation were identified. These scoring components were: (1) Deployment constraints, (2) usability, (3) comfort, (4) accuracy. This section discusses relevant information gathered in the literature review that is relevant to each of the four components

#### 3.2.1. Deployment Constraints

Features such as technology size, installation practicalities, cost and data streaming capability were some of the most common issues found in this category. A move away from large lab-based technology and towards smaller and cheaper technology, such as IMU sensors, is a simple solution to this problem.

#### 3.2.2. Usability

Usability issues not only concerns traditional software user experience but also considers device usability. While software user experience was rarely discussed or considered in any of the reviewed works, the clinicians ability to review and assess relevant data quickly and easily is vital for the success of any remote rehabilitation system. It has already been shown that clinicians fail to adopt clinic-based technologies due to usability based issues [11] and it stands to reason that clinicians will also fail to adopt remote rehabilitation technology if they are not usable. Another aspect of usability that also needs to be considered is usability from the patients aspect. The reviewed works show balance assessment technology can operate without the need for patients to interact with software. However, in remote settings there will be a requirement for patients to interact with physical devices. A common usability issue found in the reviewed works related to users needing to don and doff the device. We highlight potential issues related to donning and doffing such as hard to reach locations like the back, donning/doffing multiple devices and donning/doffing devices that require placement at specific anatomical locations. Considering the reduced range of motion and dexterity of older adults, these issues in relation to donning and doffing are of particular concern. Utilising easy to reach mounting locations and simple to use strap mechanisms are some potential solutions that can be employed to solve these issues.

#### 3.2.3. Comfort

Comfort is another key barrier that will affect patient adherence levels in any wearable sensing application. It is vital that the wearable device does not impair mobility and independence [18]. Physical discomfort can be caused by use of large/multiple sensors, unnatural sensor locations, use of poor mounting materials and sensors and/or mounting materials that inhibit body movement. Social comfort refers to how socially comfortable a user is in wearing a device. It can be crucial in the long term adoption of wearable sensors and relates to users perceptions of the device’s aesthetics [46]. While comfort was not explicitly assessed in any of the reviewed works, the use of a small wearable IMU sensor mounted on a waist mount elastic belt which is worn around the hip was successfully used in two studies to record data for up to eight days with 51 and 319 participants aged 65 and over [30,31]. Good adherence levels for 350+ older adults over a one week period is encouraging and indicates that physical and social comfort was acceptable.

While considering deployment, usability and comfort, we therefore conclude that a single small wearable IMU is the technology most likely to work in remote rehabilitation setting. If worn around the waist, using a belt based attachment, or on the wrist, using a watch like attachment, IMUs could potentially be accepted by older adults for common daily usage. It was observed that IMU sensors were the most used sensors in the reviewed works employed to assess balance and that they are located preferably near the centre of mass, on the lower back. If only one sensor is used, measurements can be extremely effective if an IMU sensor is located either at the head or at the lower back, as can be observed in [31]. Here, research focused on demonstrating data from a single accelerometer located in the trunk whilst performing daily life gait can be predictive of falls (0.66–0.72 for time to the first fall and 0.69–0.76 for the second fall).

#### 3.2.4. Accuracy

Accuracy is another issue we highlight as a potential barrier to adaption in remote rehabilitation settings. While deployment, usability and comfort are all issues relating to end user accepting and adopting the technology, issues of generalised performance relate to differences between system performance in experiments and system performance in real-world conditions. Specifically, we note that it is likely that performance reported in a number of reviewed works are over-optimistic due to issues with training and evaluation procedures. It is vital that performance metrics are representative of how a system will perform when deployed in real-world settings. In order to report performance that will generalise to new unseen participants, it is suggested that researchers use practices such as a single holdout test set and perform model and feature selection only on training data. A major issue for researchers in accurately assessing accuracy is getting a large enough sample size. It is more difficult to assess the potential of technology for real-world deployment when the sample size is small.

While it is difficult to assess the trade-off between accuracy and cost due to the limited information on the cost of some devices reviewed, it appears that there is a correlation between device accuracy and device cost.

### 3.3. Objective Assessment Rubric

As defined in the methodology, in order to develop an assessment rubric, weighting factors are assigned to each of the scoring components as defined by independent raters. Results of the independent weighting process, using the median score of three expert raters, were: Deployment Constraints =0.1, Usability =0.35, Comfort =0.2, Accuracy =0.35.

To analyse the degree of agreement between expert raters (Physios 1–3), we calculated Cohen’s Kappa regarding the perceived priority order of the components comprising the Rubric. Physios 2 and 3 had a Kappa (κ) of 1, corresponding to a perfect agreement, which was significant below 0.05 level (0.01). The order given by them was (1) usability, (2) generalised performance, (3) comfort, (4) deployment constraints. Physios 2 and 3 disagreed with Physio 1 in generalised performance (1) and usability (2). This Kappa (0.333) was weak and not significant (0.248).

In addition to calculating weighting factors for the rubric, assessment criteria need to be defined based on results of scoring component analysis and based on results of the literature review. For each of the three human factor-based components (Deployment, Usability, Comfort), a set of assessment criteria was defined which should be considered when evaluating the feasibility of the component in real-world remote rehabilitation settings. For Deployment, the criteria relates to portability, data collection infrastructure and consumer availability. For the usability component, criteria such as peripheral equipment, software usability and battery/power requirements must be considered. Moreover, for the comfort component, sensor invasiveness and donning/doffing must be considered. When accuracy is also included, a total of nine criteria were identified: (1) Portability, (2) data collection infrastructure, (3) consumer accessibility, (4) peripheral equipment, (5) software usability, (6) power consumption, (7) sensor invasiveness, (8) donning and doffing and (9) accuracy.

The score for each component is calculated using the average scores of the criteria within that component. For example, scores for portability, data collection infrastructure and cost should be averaged to calculate a rating for the Deployment component. If a criterion cannot be assessed, due to unavailable information, that score should be marked as null and the average calculation should only consider available scores. After scores are calculated for the four components, the overall score is calculated by multiplying each score by its weighting factor and summing the four weighted scores. Results of the rubric development process are shown in Table 2. The rubric includes the four scoring components and associated weights and a description of each criterion.

### 3.4. Objective Assessment Results

In this section, results of the objective assessment tool applied to the 23 studies identified in the literature are presented. Two authors, who did not contribute to defining the assessment tool weightings, independently rated each article under each of the nine assessment criteria. For each article, component scores for raters 1 and 2 were averaged to calculate an average score for each of the four scoring components. An overall score was then calculated by applying the weighting factor to each component score and then summing the four weighted scores. Table 3 details the average score assigned by the rater for each component as well as the total weighted score. Note that the table is sorted with highest scoring articles appearing at the top of the table.

## 4. Discussion

Technology supported balance assessment techniques offer a number of advantages over traditional non-standardised and standardised clinical assessments such as BBS and TUG. Technology can ensure that the administration of the assessment is objective and that results are quantitative. Technology supported balance assessment also possesses desirable characteristics that give it potential to be used in remote settings without the need for patients to travel to clinics and without the need for clinicians to administer. For example, the literature shows that longer periods of monitoring may lead to more accurate balance assessments. If indeed longer periods of monitoring are required, then sensor-based systems in remote settings are the most feasible option.

A literature review revealed that balance assessment technology for older adults is mainly focused on understanding the cause of poor balance, identifying falls or predicting risk of falls in the future. All 23 reviewed research studies focused on the validity, reliability and accuracy of the technologies, sensor systems, and data models. In contrast, very few of these studies had a specific focus on the human factors of the sensor systems such as comfort or practicality. However, for a rehabilitation sensor to be feasible in real-world conditions, it must be comfortable to wear or mounted in a fixed position, accurate enough to identify compensatory movements, affordable, widely available, not require calibration, and be robust with regard to patient characteristics [42].

Using knowledge gained from the literature review, this work defined four key requirements needed for remote rehabilitation applications. We propose that these four requirements should be considered when evaluating remote rehabilitation applications such as balance assessment technologies. In applying the proposed assessment tool to the literature, results revealed that works utilising either a single accelerometer device [30,31], a smartphone embedded sensor [39], or an insole based device [26] scored well in all criteria based on average scores assigned by two independent raters. While works utilising multiple different sensors generally scored well when just measuring accuracy, once Deployment, Usability and Comfort factors are considered, these works generally scored much lower.

It is clear, based on a number of works scoring well in our objective assessment rubric, that technology is advancing in a positive direction. Sensors, particularly small accelerometer based devices, show excellent potential to be utilised to assess balance in remote rehabilitation settings. By scoring well in most criteria, it is possible to put human factors at the core of the design without compromising significantly on accuracy.

In the future, as the research area continues to grow, it is important that researchers give human factors a more central focus if we are to realise the potential of sensor based balance assessment in remote settings. Retrospectively improving the usability of a system that, for example, achieved excellent accuracy but requires the use of impractical, uncomfortable and difficult to use systems is an extremely challenging task. Rather than researchers starting with accuracy as the main goal, consideration should be first given to the human factors associated with any selected sensors and assessment protocols. Research goals should then focus on increasing the accuracy of these more practical and usable systems. We propose that researchers should consider our proposed assessment tool, based on the 9 defined criteria, when designing and evaluating new technology.

This work formed part of SENDoc and influenced other elements of the project which involve testing sensors on elderly people in clinic settings, and in their communities in the northern periphery of Europe. This includes collecting views of using the sensors.

## 5. Conclusions

This work discussed the importance of accurate and objective clinical balance assessment methods. A review was conducted on the state of the art related to technology enabled balance assessment methods and their potential to be utilised in remote rehabilitation settings. As part of the literature review, a set of six key themes were identified and discussed. Results of the literature review led to the creation of an objective assessment rubric based on four scoring components comprising a total of 9 assessment criteria. Each criterion was identified based on its importance in real-world deployment of remote rehabilitation technology, and a weighting to each criterion was decided through three independent physiotherapists.

Most clinical balance tests are subjective, but the addition of technology for monitoring the process enhances objectivity and changes it into a quantitative assessment. Even with the increased accuracy that sensors can bring to standardised tests, it was observed that non-standardised methods of assessing balance are still frequently used in clinical settings due to their simplicity in administering. In order for technology to be used to support remote assessment of balance, it must therefore be simple and easy to administer for clinicians. Ease of use must also be considered from the patients point of view. In particular the technology must not impair mobility or independence for the patient. Therefore, by supplying a methodology to facilitate a more objective assessment of potential solutions for remote rehabilitation, we can ensure that barriers to real-world adaption, such as usability, are considered much earlier in the design.

It was observed that further research in other usability features of the technology or wearable sensor systems, such as the practicality and comfort, is required in order to better understand potential barriers to adaption in home rehabilitation. Research studies have a tendency to only focus on the accuracy and the validity/reliability. Considering human factors at research conception is key to progress. Doing so will ensure the development of more practical systems which will be more likely to be adopted into real-world rehabilitation settings and thus have a positive impact on a person’s quality of life.

As the number of commercially available sensor systems are increasing, it has become increasingly more difficult for clinicians to identify appropriate sensor systems for specific use cases including balance assessment. While considering sensors and technologies, it is vital that clinicians and researchers have objective methodologies available to evaluate different balance assessment technologies available. This paper proposes an objective assessment tool to evaluate the different sensor systems for their potential to be adapted in a remote rehabilitation setting. When applying the tool to the literature, it is clear that there is significant potential for technology to be deployed in remote rehabilitation settings. However, there still remains some issues which should be the focus of future work. These issues relate to an increased focus on objectively assessing the usability of systems and supporting software.

## Figures and Tables

**Table 1:** Balance assessment using technology literature review summary.

Research	Rational/Focus on and Cohort	Sensors/Technology Used and Deployment	Findings	Human Factors
Walsh et al. (2011) [19]	To assess the validity and reliability of a portable quantitative balance measurement technology compared to the force plate (the gold standard) Cohort: 2 participants (1M, 29; 1F, 22)	AMTI (Advanced Mechanical Technology) force plates Tactex high density (HD) pressure sensor mat SHIMMER IMU sensor (used for syncing pressure sensor and force plate systems) Participant stands on pressure mat wearing IMU sensor	• The two Berg Balance Scale (BBS) estimates of each participant in successive trials, using lasso model with automatic segmentation of data, have a mean absolute error of 1.44 points • The proposed technique predicts accurate functional balance of the elderly people and has the potential to act as a surrogate of BBS test. However, the cohort is too small, only 2 participants	• Portable alternative to a force plate system means there is potential for remote use • Laptop is used to collect data and needs trained professional to operate, making system appropriate for community care or use in care facilities • Additional IMU unit required for syncing adds complexity to the setup
Greene et al. (2012) [20]	Using body worn sensor data to predict falls in community dwelling Cohort: 226 participants (62M; 164F; 60+ years old; mean 71.5 ± 6.7)	Shimmer sensors (IMUs) mounted on the left and right shanks to quantify gait and lower limb movement while performing the TUG test	• Results obtained through cross validation yielded a mean classification accuracy of 79.69% (mean 95%, CI: 77.09–82.34) • The quantification of movement during the TUG test using sensors could lead to a robust method assessing future fall risk • Results were significantly (*p* < 0.0001) more accurate than those obtained using BBS and manual TUG	• The setup is deemed as quite unobtrusive due to two sensor setup • The system is deemed as easy to don/doff due to shank sensor location • Shimmer units have limited storage space and require commercial grade software to analyse results
Kearns et al. (2012) [21]	Fall prediction and standardized gait and balance assessments: Focused on analysing the variability in voluntary movement paths of assisted living facility (ALF) residents. The authors observed greater movement variability in the week preceding a fall. Cohort: 69 participants (16M; 53F; mean age 76.9 ± 11.9)	Tele-surveillance technology 4 room mounted sensors and one participant worn ubisense compact tag	• Logistic regression analysis revealed odds of failing increased 2.548 (*p* = 0.021) for every 0.1 increase in fractal D, and a having a fall in the prior year increased odds of falling (OR 0.976 *p* = 0.08) but it was no significant • Fallers had more variable stride to stride velocities and required more activities of daily living assistance	• The wrist-mounted compact tag worn by participants was found to be uncomfortable by 38% of the cohort • The sensor was able to be mounted to participants walking aid without affecting results or accuracy • The trial lasted for one year, thus technology deemed appropriate for long term use • The expense and setup of the system (4 room mounted tracking sensors) makes it more suitable for a carehome environment
Barelle, Houel and Koutsouris (2014) [22]	Focused on creating a falls model based on cluster analysis (accessible biomechanics predictors). The study focusses on assessing whether or not there is gait impairment, which is correlated with loss of physical function and fall risk. Cohort: 18 participants in 3 groups (6 healthy elders (3F, 3M; 65+); 6 fallers (3F, 3M; 65+); 6 healthy control (3F, 3M; 24–26))	VICON motion capture system composed of 8 Infrared (IR) video cameras was used to track 33 external reflective markers located on the participant	• ANOVA used to analyse differences between healthy controls, healthy elders and fallers. In terms of stride to stride parameters and Active ROM (AROM) for the hip, the knee and the ankle • 8% decreased of the knee AROM in the elderly is found compared to the young • Only significant differences appear in stride length and step length as well as hip and knee AROM between young and elderly including fallers. These deviation remains with comparing the three groups with *p* < 0.07	• Vicon system deemed difficult and cumbersome to don/doff as 33 markers are required to be worn • 8 cameras required for system to operate means system is not easily transportable or transferable thus inappropriate for remote use • Expert knowledge required to set up and operate system • Bulky clothes cannot be worn when using system
Reynard et al. (2014) [23]	Early gait stability index to prevent falls (Assessing Local Dynamic Stability (LDS) to small perturbations) Cohort: 83 patients with mild to moderate gait disorders (35F, 48M; mean age 44 ± 14) 40 healthy control (20F, 20M; mean age 40 ± 9)	Physilog system (IMUs) by GaitUp was used to record trunk (at the level of the L3–L4) accelerations along three axes: Medio-lateral (ML), vertical (V) and antero-posterior (AP) Single IMU sensor attached to trunk with elastic belt	• The Local Dynamic Stability (LDS) measured in short over ground walking tests seems sufficiently reliable. • LDS assessed along the medio-lateral axis offered the highest repeatability and discriminative power. Intrasession repeatability in the patients was 0.89 and the smallest detectable difference was 16%. • LDS was substantially lower in the patients than in the controls (33% relative difference, standardized effect size 2.3)	• The Physilog system is deemed easy to don/doff due to one sensor setup with elastic belt • System is lightweight so would not be cumbersome to wear for extended periods of time • Bespoke software required to analyse results
Finkelstein and Jeong (2015) [24]	Assessing autonomic balance by analysing the activity of the autonomous nervous system. This is achieved through analysing heart rate variability (HRV) during a cycling exercise Cohort: 5 participants (healthy)	BN-RSPE, BIOPAC Systems, Wireless electrocardiogram (ECG) device 9 pre-gelled and disposable ECG electrodes worn on the chest (LL Electrode series, Lead-Lok)	• Discriminant function analysis was conducted to investigate a potential value of discrimination among elders and patients with heart diseases • When cross-validated classification was performed that was using the leave-one-out method, overall 86.7% of originally grouped cases were correctly classified	• The wireless ECG is deemed difficult to don/doff due to the 9 electrode setup which requires precise positioning on chest. • The ECG electrodes require gel to make contact with human skin–this is invasive and may not be perceived as comfortable by patients. • Health care professional required to setup ECG sensors and take readings thus system is not suitable for remote use
Ràbago, Dingwell and Wilken (2015) [25]	Determining the between-session reliability and minimum detectable change values of temporal-spatial, kinematic variability, and dynamic stability measures during three types of perturbed gait (used to identify dysfunction associated with gait instability) Cohort: 20 participants (young healthy adults)	Vicon Motion Systems composed of 24 IR cameras to track 57 reflective markers located on hand, arm, head, trunk, pelvis, thigh, leg and foot segments	• Participants during session 1 exhibited a significant 8% increase (*p* = 0.001, d = 0.35) in mean speed walking compared to unperturbed participants with no significant change in speed walking • Participants walked at an average speed of 1.20 ± 0.04 m/s across all walking conditions. In response to all perturbation conditions • All temporal-spatial, kinematic variability and dynamic stability measures demonstrated fair to excellent between-session reliability.	• Vicon system deemed difficult and cumbersome to don/doff as 57 markers are required to be worn • 24 cameras required for system to operate means system is not easily transportable or transferable thus inappropriate for remote use • Expert knowledge required to set up and operate system
Ayena et al. (2016) [26]	Improving and facilitating the methods to assess risk of falling at home among elders by computing the risk of falling in real time daily activities Cohort: 29 participants (17 elders, 59–79 (10 healthy, 7 Parkinson’s Disease); 12 healthy adults)	Custom made instrumented insole with Bluetooth capability connected to a Smartphone. This device comprises a set of sensors such as accelerometers (located in electronic board), force sensors and bending variable sensor Insoles placed inside participants shoes	• Results suggest that there is a relationship between OLST score and the risk of falling based on centre of pressure measurement • The risk of falling depends on type of ground (ground properties such as compliance and coefficient of friction) • The main finding of this work is that this model could be used to simulate the balance capability and could be implemented inside the embedded microcontroller of the insole in real-time	• Initial calibration required in clinic using tether-release system • Insole is designed to be worn in every day life; home monitoring is dealt with using a mobile phone app serious game for exercise • Insole deemed easy to don/doff, pairs to mobile phone, and has been designed with home rehabilitation in mind
Hong et al. (2016) [27]	Assessing the stability of human postural balance by using a force plate Cohort: 40 participants split in two groups group 1: 20 participants (10F, 10M; 65–73 years old; mean age 68.7 ± 2.96) group 2: 20 participants (10F, 10M; 18–24 years old; mean age 20.1 ± 1.29)	Force plate (custom made-Piezo electric force transducers were positioned in 4 corners) Participant stands on plate	• The proposed features are not only robust to intertrial variability but also more accurate than one of the most effective COP features and two recently proposed COM features in classifying the older and younger age groups • The proposed approach reduces the force sensor requirements from 3D to 1D, substantially reducing the cost of the force plate measurement system	• Force plates are not suitable for home/remote rehabilitation. They are a research grade device intended for use in clinics. They are very expensive, large and cumbersome. • The approach would be difficult and expensive to be transferred to a remote or home-based setting
Howcroft, Lemaire, and Kofman (2016) [28]	Gait-based sensor assessment for fall-risk, which involves identifying the sensors, the location and modelling method Cohort: 100 participants (mean age 75.5 ± 6.7; 76 non-fallers, 24 fallers)	Pressure-sensing insoles (F-Scan 3000E, Tekscan) and tri-axial accelerometers (X16-1C, Gulf Coast Data Concepts) IMUs were worn at the posterior head, posterior pelvis, and lateral left and right shanks (just above an ankle with a band)	• The best performing model was a multi-layer perceptron neural network with input parameters from pressure-sensing insoles and head, pelvis, and left shank accelerometers (accuracy = 84%, F1 score = 0.600, MCC score = 0.521) • Head sensor-based models had the best performance of the single-sensor models for Single-Task (ST) gait assessment • ST gait assessment models outperformed models based on dual-task (DT) walking or clinical assessment data	• Impractical in remote settings due to combination of head, pelvis, shank and insole mounted sensors. • Accelerometers are likely to be perceived uncomfortable and difficult to don/doff • Remote monitoring difficult due to both hardware comms limitations and only research grade software being available
Mohler et al. (2016) [29]	Using sensor-based measures of gait, balance and Physical Activity (PA) in community dwelling Cohort: 119 participants (95F, 24M; 65 years +; mean age 78.46 ± 8.4	LEGSysTM; BioSensics Five small inertial sensors are tri-axial accelerometer and gyroscope attached to the shins above ankles, thighs above knees, and lower back close to the sacrum	• Balance deficit and PA were independent fall predictors in pre-frail and frail groups. They were not sensitive to predict prospective falls in the non-frail group • Even thought gait performance deteriorated as frailty increased, gait was not a predictor of prospective falls when participants were stratified based on frailty status	• Impractical and cumbersome in remote settings due to 5 sensor setup (shins, thighs, and lower back). Correct placement of sensors tricky without expert • Accelerometers are likely to be perceived uncomfortable and difficult to don/doff due to quantity and placement • Research grade software required to analyse results for feedback
van Lummel et al. (2016) [30]	Assessing the quality of life of an individual (health status, functional status and physical activity) related to Sit to Stand test (STS) Cohort: 51 female participants 64 years + (mean age 83 ± 6.9)	IMU sensor Dynaport Hybrid was used (durations, sub durations and kinematics), physical activity was followed for 1 week with an activity monitor (laying, sitting, standing and locomotion) One IMU sensor worn in an elastic belt worn around the waist and fixed over the lower back	• The manually recorded STS test was not significantly associated with the health status (*p* = 0.475) and functional status (*p* = 0.055), while the instrumented STS times were both (*p* = 0.009). • Duration’s of the Dynamic sit to stand phase of the instrumented STS showed more significant associations with health status, functional status and daily physical activity (all *p* = 0.001) than the static phases standing and sitting (*p* = 0.043–0.422)	• Deemed easy to don/doff due to one sensor setup • Worn by participants in study for 1 week so deemed comfortable for use over long periods of time • Commercial software required to process signals and analyse results
van Schooten et al. (2016) [31]	Assessing physical activity and daily life gait quality (in terms of stability, variability, smoothness and symmetry) and determine their predictive ability for time-to-first-and second falls Cohort: 319 participants (163F, 156M; 65–99 years old; mean age 75.5 ± 6.9)	Dynaport Move Monitor by McRoberts tri-axial accelerometer One IMU sensor placed in an elastic belt and worn around the waist, fixed over the lower back (fifth lumbar vertebra, L5)	• Gait characteristics-walking speed, stride length, stride frequency, intensity, variability and smoothness, symmetry and complexity-were often moderately to highly correlated (>0.4) • The cross-validated prediction models had adequate to high accuracy (time dependent AUC of 0.66–0.72 for time to first fall and 0.69–0.76 for second fall) • Daily life gait quality obtained from a single accelerometer on the trunk is predictive for falls	• Deemed easy to don/doff due to one sensor setup • Worn by participants in study for 8 days so deemed comfortable for use over long periods of time • Commercial software required to process signals and analyse results
Zihajehzadeh and Park (2016) [32]	Walking speed is assessed to study human health status through IMU sensors in the wrist. Walking speed variation or change in its trajectories can be linked to cognitive impairment, multiple sclerosis, Parkinson’s disease, risk of falls, kidney disease and adverse effects of aging (disability and hospitalization) Cohort: 15 participants (9M, 6F; mean age 27 ± 4)	Xsens MTiG-700 IMU and the Global (accelerometer, magnetometer and gyroscope) worn on the left wrist The Positioning System (GPS) is only used in the outdoor walking trial of this study	• Results show that the use of the pca-acc variable can significantly improve the walking speed estimation accuracy when compared to the use of raw acceleration information (*p* < 0.01) • When the Gaussian process regression is used, the resulting walking speed estimation accuracy and precision is about 5.9% and 4.7%, respectively	• Deemed easy to don/doff due to one sensor wrist-worn setup • No tricky sensor locating required as sensor is wrist-worn on a piece of elastic • Study with participants lasted for 12 min however comfort is not deemed to be an issue as wearing IMU is comparable to wearing a watch • Research grade software required to analyse results
Kikkert et al. (2017) [33]	Falls prediction - Dynamic parameters of gait-gait control (balance) Cohort: 61 participants (41F, 20M; 70 years+)	Dynaport1 MiniMod, McRoberts A tri-axial accelerometer attached to the lower back at the level of the third lumbar spine segment to measure medio-lateral (ML) and anterior-posterior (AP) trunk accelerations	• Classification accuracy of models (1), (2) and (3) were 0.86, 0.90 and 0.93. Specificity in the third model was 80% in comparison to 72% and 60% reached by models (2) and (1), respectively. Sensitivity values were 92%, 89% and 92% for models (1), (2) and (3), respectively • Results show that combining gait-speed and speed related measures with dynamic gait measures will increase specificity and thus classification accuracy	• Deemed easy to don/doff due to one sensor setup • Worn by participants in study for a 160m walk, however same sensor has been worn in other studies for >1 week. Comfort therefore unlikely to be an issue • Specific locating at third lumbar spine segment means training would be required for users to correctly position while at home • Commerical software required to process signals and analyse results
Ocampo et al. (2017) [34]	Analysing muscle fatigue for enhancing performance of existing fall detection systems Cohort: 20 healthy participants	Surface ElectroMyoGraphy (SEMG) for muscle fatigue information and accelerometer (ACC) sensors 5 SMEG sensors on leg and 2 Accelerometers worn	• Results showed that the combination of SEMG and ACC data have relatively increased the accuracy of fall detection systems • Linear regression was used to estimate the CT and EMG MPFT values. Two separate paired-samples *t*-tests were used to compare the mean absolute and %MVIC values for CT and EMG MPFFT. In addition, a Pearson correlation was used to determine the relationship between the absolute CT and EMG MPFFT values. An alpha of *p* < 0.05 was considered statistically significant	• The system is deemed difficult to don/doff due to complex 7 sensor setup • The sensors need precise positioning and thus a trained professional to assist • The SMEG sensors are attached to the body using elastic bands. This may not be comfortable for extended periods of time • Commercial grade software required to process data
Shahzad et al. (2017) [35]	Obtaining an objective, cost-effective, and unsupervised method to obtain functional balance and mobility assessment-based fall-risk of community-dwelling older adults Cohort: 23 participants (16F, 7M; 60+ years old; mean age 72.87 ± 8)	Shimmer-Single triaxial accelerometer sensor attached on the lower back between the L3-L5 vertebrae by means of elasticised bandages to measure the trunk acceleration	• High correlation (*p* = 0.90) and low root-mean-square error (1.66) was observed between the two estimates of each subject • The average value of the estimates achieved low RMSE error (2.78) with respect to clinical BBS score, which demonstrate that the proposed technique predicts accurate functional balance of the elderly people and has the potential to act as a surrogate of BBS test in an unsupervised setting • The elderly people can assess their balance any time at-home without the need of physiotherapist/expert	• Deemed easy to don/doff due to one sensor setup, although training would be required to ensure correct positioning was achieved • Single sensor is neither cumbersome nor weighty, so deemed acceptable to wear for longer periods of time. Some comfort issues may arise over prolonged use of elastic belt • Sensors stream data to bespoke MATLAB application over bluetooth, and a trained observer annotates the datastream in this particular experiment.
Jafari et al. (2018) [36]	To understand the mechanism behind the reduced ability to maintain balance in any posture or activity. Studying the performance of the central nervous system (CNS) as a controller of the body, while maintaining the balance in some postures or activities Cohort: 45 participants (18M, 27F; 70+ years old; mean age 75.2 ± 4.5)	Qualisys Oqus 4 system: An Optic system with eight cameras for 3D motion capture): A full body marker model with a total of 60 pieces of 10 mm round reflective markers Noraxon DTS 16 channel: A wireless system for EMG collection	• Results show that the model is capable to adapt to the changes in the input signals and predicts the normalized and rectified EMGs with high accuracy (Average RMSE = 0.06 V for all subjects in the test data set) • The overall scheme can adapt to physical body characteristics of different subjects, the changes of multiple sensory inputs and successfully predict the muscle activity based on the optimum number of sensory inputs	• System deemed very difficult to don/doff due to 4 sensor EMG sensor setup which requires precise positioning and gels applied • Optic system requires 60 markers placed on the body, again with precise positioning meaning a trained professional is required • The cost and complexity of this systems means it is not suitable for home use
Levy, Thralls, and Kviatkovsky (2018) [37]	Examining the current validity and 3-day test-retest reliability of the Balance Tracking System Cohort: 96 participants in total (57F, 39M; mean age 73.5 ± 7.79)	BTrackS–a portable force plate. Participant stands on force plate.	• BTrackS demonstrated good validity using Peason product moment correlations (r > 0.90). • Test-retest reliability using ICCs was excellent (0.83) and calculated MDC for Eyes Open (9.6 cm) and Eyes Closed (19.4 cm) conditions, and suggested that postural sway changes of these amounts are meaningful • BTrackS has the potential to identify meaningful changes in balance that may warrant intervention	• BTrackS is portable, affordable, and lightweight sensor plate intended for clinical use • The system connects to a laptop via USB thus requiring a medical professional on site to operate • Interpreting data recorded requires expert knowledge and thus is not appropriate for home user use.
Virmani et al. (2018) [38]	Assess gait and balance in healthy non-fallers Cohort: 75 participants (42F, 33M; aged 21–79; mean age 46.9 ± 17.1)	Zeno Walkway, Prokinetics (PKMAS) Pressure sensor mat which participant walks on.	• Stepwise multivariate analysis of all 31 parameters assessed from three different gait paradigms showed weak but significant correlations in age with (a) stride to stride variability in (b) integrated pressure of footsteps and (c) mean stride length on dual task and (d) mean step width on tandem gait (R2 = 0.382, t = 2.26 *p* = 0.026 • There were weak but significant age-related changes in objective measures of steady state gait and balance. Impaired contrast sensitivity, not visual acuity, correlates with decreased stride-length and mean steady state stride length was not a parameter in the final model. As one ages, there is more variability in the pressure applied while stepping and the length of each stride	• Zeno Walkway $20^{\prime }\times 4^{\prime }$ pressure sensor impregnated mat intended for clinical use • Size and cost of device not suitable for home use • Research grade Protokinetic Movement Software required for analysis
Coni et al. (2019) [39]	Apply factor analysis of sensor-based physical capability assessment to transform a battery of sensor-based functional tests into a clinically applicable assessment tool Cohort: 304 participants (141M, 163F; 65–98 years old; mean age 80.9 ± 6.4)	Galaxy SII or Galaxy SIII, Samsung: Accelerometer range ±2 g and gyroscope, was worn at the lower back (fifth lumbar vertebra, L5), taken as reference of the body COM, by means of an elastic waist belt. A custom Android application was used for recording tri-axial inertial signals (Anteroposterior, AP, Mediolateral, ML, Vertical, V) from the embedded sensors	• Instrumented tests provided 73 sensor-based measures, out of which Exploratory Factor Analysis identified a fifteen-factor model which is suitable for physical capability assessment of older adults • EFA reduced the number of sensor-based measures taken from instrumented functional tests and find domains with clear functional meaning • The research shows that instrumented functional testing has the potential to advance the quality of current mobility assessments	• Deemed easy to don/doff due to single device setup • Only worn by participants in study for a 7 m walk and 5 STS tests. Mobile phone likely to feel heavy to wear in an elasticated belt for a prolonged period of time • Custom android app records accelerometer measurements and processed afterwards using MATLAB
Tang et al. (2019) [40]	Estimating Berg Balance Scale and Mini Balance Evaluation System Test Scores Cohort: 30 participants (13M, 17F; mean age 76 ± 10.5)	Custom made pressure sensitive insole comprising three pressure sensors, were located inside of each participant’s shoe (Bluetooth communication devices were clipped outside of the shoe) The accelerometer ADXL330 from Analog devices was worn on the hip (worn in a pouch)	• The results show that the wearable sensor system has a capability to estimate the Berg Balance Scale and Mini Balance Evaluation System Test scores with absolute mean errors and standard deviations 6.07 ± 3.76 and 5.45 ± 3.65, respectively • The results demonstrate high agreement with falls history based risk assessment	• The insoles are deemed easy to don/doff and have been designed to fit in participants own shoes • The hip-worn accelerometer is also deemed easy to don/doff and position correctly due to use of pouch • Data from sensors collected on smartphone and processed offline

**Table 2:** Grading Rubric.

Category	Factor	Description	Score 1–5	Weighting Factor
	Portability	How easy is the technology to move to a persons home?		
	Data collection infrastructure	Does the technology come with remote data access to support clinician remote access?		0.10
Deployment Constraints	Consumer Accessibility	Is the technology affordable and widely available?		
Usability	Peripheral Equipment	Is additional supporting hardware/technology required for operation?		
	Software Usability	Is the supporting software user friendly for patient? (older adults)		0.35
	Power Consumption	Does the device support long term [+1 week] data recording between charges?		
	Invasiveness of sensors	Are sensors comfortable or do they restrict physical activity?		0.2
Comfort	Donn/Doffing	Are the sensors easy to put on and off for the patient?		
Performance	Accuracy	Ability to perform measurements within an acceptable error rate?		0.35

**Table 3:** Average Assessment Scores.

Paper and Technology	Pre-Weighted Component Scoring				Weighted Score
	Deployment	Usability	Comfort	Performance	
[31] Dynaport MM (accelerometer)	0.633	0.633	1.000	0.800	0.765
[26] Smart insoles	0.833	0.667	0.850	0.700	0.732
[39] Mobile phone accelerometer	0.867	0.633	0.650	0.800	0.718
[30] Dynaport Hybrid (IMU)	0.600	0.633	0.950	0.700	0.717
[37] Force plate	0.567	0.533	0.950	0.800	0.713
[23] Physilog (acceletometer)	0.633	0.633	0.650	0.800	0.695
[27] Force plate	0.433	0.500	0.800	0.900	0.693
[20] 2 Shimmer sensors (accelerometer)	0.633	0.633	0.600	0.800	0.685
[33] Dynaport1 Minimod (accelerometer)	0.667	0.667	0.600	0.700	0.665
[32] wrist-worn IMU	0.633	0.533	0.900	0.600	0.640
[35] Shimmer (accelerometer)	0.767	0.533	0.650	0.700	0.638
[21] Tele-surveillance	0.300	0.600	0.750	0.700	0.635
[40] Smart insoles and 1 accelerometer	0.700	0.633	0.650	0.600	0.632
[38] Pressure mat	0.433	0.533	0.750	0.700	0.625
[19] Pressure mat and Shimmer	0.433	0.500	0.800	0.700	0.623
[36] Qualisys (8 cameras)	0.333	0.533	0.250	1.000	0.620
[29] LEGSys (5 accelerometers)	0.633	0.500	0.300	0.900	0.613
[25] Vicon	0.267	0.533	0.300	0.900	0.588
[22] Vicon	0.267	0.500	0.500	0.800	0.581
[28] Smart insoles and 4 accelerometers	0.600	0.500	0.300	0.800	0.575
[34] 5 SEMG and 2 acceleromters	0.433	0.533	0.400	0.700	0.555
[24] Wireless ECG	0.500	0.433	0.300	0.700	0.506

## References

[1] Gomes M., Figueiredo D., Teixeira L., Poveda V., Paúl C., Santos-Silva A., Costa E. (2016). Physical inactivity among older adults across Europe based on the SHARE database. Age Ageing.

[2] Munoz Esquivel K., Nevala E., Alamäki A., Condell J., Kelly D., Davies R., Heaney D., Nordström A., Åkerlund Larsson M., Nilsson D. (2018). Remote Rehabilitation: A solution to Overloaded & Scarce Health Care Systems. Trends Telemed. E-Health.

[3] Vuononvirta T., Timonen M., Keinänen-Kiukaanniemi S., Timonen O., Ylitalo K., Kanste O., Taanila A. (2011). The compatibility of telehealth with health-care delivery. J. Telemed. Telecare.

[4] Mancini M., Horak F. (2010). The relevance of clinical balance assessment tools to differentiate balance deficits. Eur. J. Phys. Rehabil. Med..

[5] Barnea R., Weiss Y., Abadi-Korek I., Shemer J. (2018). The epidemiology and economic burden of hip fractures in Israel. Isr. J. Health Policy Res..

[6] Wang Q., Markopoulos P., Yu B., Chen W., Timmermans A. (2017). Interactive wearable systems for upper body rehabilitation: A systematic review. J. NeuroEng. Rehabil..

[7] Klaassen B., van Beijnum B.J., Held J., Reenalda J., van Meulen F., Veltink P., Hermens H. (2017). Usability evaluations of a wearable inertial sensing system and quality of movement metrics for stroke survivors by care professionals. Front. Bioeng. Biotechnol..

[8] Langley F.A., Mackintosh S.F. (2007). Functional Balance Assessment of Older Community Dwelling Adults: A Systematic Review of the Literature. Internet J. Allied Health Sci. Pract..

[9] Southard V., Dave M., Davis M.G., Blanco J., Hofferber A. (2005). The Multiple Tasks Test as a predictor of falls in older adults. Gait Posture.

[10] Lemay J.F., Nadeau S. (2010). Standing balance assessment in ASIA D paraplegic and tetraplegic participants: Concurrent validity of the Berg Balance Scale. Spinal Cord.

[11] Sibley K., Inness E., Straus S., Salbach N., Jaglal S. (2013). Clinical assessment of reactive postural control among physiotherapists in Ontario, Canada. Gait Posture.

[12] Gordt K., Gerhardy T., Najafi B., Schwenk M. (2017). Effects of Wearable Sensor-Based Balance and Gait Training on Balance, Gait, and Functional Performance in Healthy and Patient Populations: A Systematic Review and Meta-Analysis of Randomized Controlled Trials. Gerontology.

[13] Díaz S., Stephenson J.B., Labrador M.A. (2019). Use of Wearable Sensor Technology in Gait, Balance, and Range of Motion Analysis. Appl. Sci..

[14] Leirós-Rodríguez R., García-Soidán J.L., Romo-Pérez V. (2019). Analyzing the use of accelerometers as a method of early diagnosis of alterations in balance in elderly people: A systematic review. Sensors.

[15] Hubble R.P., Naughton G.A., Silburn P.A., Cole M.H. (2015). Wearable sensor use for assessing standing balance and walking stability in people with Parkinson’s disease: A systematic review. PLoS ONE.

[16] Porciuncula F., Roto A.V., Kumar D., Davis I., Roy S., Walsh C.J., Awad L.N. (2018). Wearable Movement Sensors for Rehabilitation: A Focused Review of Technological and Clinical Advances. PM R.

[17] Ghislieri M., Gastaldi L., Pastorelli S., Tadano S., Agostini V. (2019). Wearable inertial sensors to assess standing balance: A systematic review. Sensors.

[18] Gövercin M., Költzsch Y., Meis M., Wegel S., Gietzelt M., Spehr J., Winkelbach S., Marschollek M., Steinhagen-Thiessen E. (2010). Defining the user requirements for wearable and optical fall prediction and fall detection devices for home use. Inform. Health Soc. Care.

[19] Walsh L., Greene B.R., McGrath D., Burns A., Caulfield B. Development and validation of a clinic based balance assessment technology. Proceedings of the 2011 Annual International Conference of the IEEE Engineering in Medicine and Biology Society.

[20] Greene B.R., Doheny E.P., Walsh C., Cunningham C., Crosby L., Kenny R.A. (2012). Evaluation of Falls Risk in Community-Dwelling Older Adults Using Body-Worn Sensors. Gerontology.

[21] Kearns W.D., Fozard J.L., Becker M., Jasiewicz J.M., Craighead J.D., Holtsclaw L., Dion C. (2012). Path Tortuosity in Everyday Movements of Elderly Persons Increases Fall Prediction Beyond Knowledge of Fall History, Medication Use, and Standardized Gait and Balance Assessments. J. Am. Med. Dir. Assoc..

[22] Barelle C., Houel N., Koutsouris D. A cluster analysis approach for the determination of a fall risk level classification. Proceedings of the 2014 IEEE 19th International Workshop on Computer Aided Modeling and Design of Communication Links and Networks (CAMAD).

[23] Reynard F., Vuadens P., Deriaz O., Terrier P. (2014). Could Local Dynamic Stability Serve as an Early Predictor of Falls in Patients with Moderate Neurological Gait Disorders? A Reliability and Comparison Study in Healthy Individuals and in Patients with Paresis of the Lower Extremities. PLoS ONE.

[24] Finkelstein J., Jeong I.C. (2015). Using heart rate variability for automated identification of exercise exertion levels. Stud. Health Technol. Inform..

[25] Rabago C.A., Dingwell J.B., Wilken J.M. (2015). Reliability and Minimum Detectable Change of Temporal-Spatial, Kinematic, and Dynamic Stability Measures during Perturbed Gait. PLoS ONE.

[26] Ayena J.C., Zaibi H., Otis M.J., Ménélas B.J. (2016). Home-Based Risk of Falling Assessment Test Using a Closed-Loop Balance Model. IEEE Trans. Neural Syst. Rehabil. Eng..

[27] Hong C.Y., Guo L.Y., Song R., Nagurka M.L., Sung J.L., Yen C.W. (2016). Assessing postural stability via the correlation patterns of vertical ground reaction force components. BioMed Eng. OnLine.

[28] Howcroft J., Lemaire E.D., Kofman J. (2016). Wearable-Sensor-Based Classification Models of Faller Status in Older Adults. PLoS ONE.

[29] Mohler M.J., Wendel C.S., Taylor-Piliae R.E., Toosizadeh N., Najafi B. (2016). Motor Performance and Physical Activity as Predictors of Prospective Falls in Community-Dwelling Older Adults by Frailty Level: Application of Wearable Technology. Gerontology.

[30] Van Lummel R.C., Walgaard S., Maier A.B., Ainsworth E., Beek P.J., van Dieen J.H. (2016). The Instrumented Sit-to-Stand Test (iSTS) Has Greater Clinical Relevance than the Manually Recorded Sit-to-Stand Test in Older Adults. PLoS ONE.

[31] van Schooten K.S., Pijnappels M., Rispens S.M., Elders P.J.M., Lips P., Daffertshofer A., Beek P.J., van Dieen J.H. (2016). Daily-Life Gait Quality as Predictor of Falls in Older People: A 1-Year Prospective Cohort Study. PLoS ONE.

[32] Zihajehzadeh S., Park E.J. (2016). Regression Model-Based Walking Speed Estimation Using Wrist-Worn Inertial Sensor. PLoS ONE.

[33] Kikkert L.H.J., de Groot M.H., van Campen J.P., Beijnen J.H., Hortobagyi T., Vuillerme N., Lamoth C.C.J. (2017). Gait dynamics to optimize fall risk assessment in geriatric patients admitted to an outpatient diagnostic clinic. PLoS ONE.

[34] Ocampo J.P.F.E., Dizon J.A.T., Reyes C.V.I., Capitulo J.J.C., Tapang J.K.G., Prado S.V. Evaluation of muscle fatigue degree using surface electromyography and accelerometer signals in fall detection systems. Proceedings of the 2017 IEEE International Conference on Signal and Image Processing Applications (ICSIPA).

[35] Shahzad A., Ko S., Lee S., Lee J., Kim K. (2017). Quantitative Assessment of Balance Impairment for Fall-Risk Estimation Using Wearable Triaxial Accelerometer. IEEE Sens. J..

[36] Jafari H., Pauelsen M., Röijezon U., Nyberg L., Nikolakopoulos G., Gustafsson T. On Internal Modeling of the Upright Postural Control in Elderly. Proceedings of the 2018 IEEE International Conference on Robotics and Biomimetics (ROBIO).

[37] Levy S.S., Thralls K.J., Kviatkovsky S.A. (2016). Validity and Reliability of a Portable Balance Tracking System, BTrackS, in Older Adults. J. Geriatr. Phys. Ther..

[38] Virmani T., Gupta H., Shah J., Larson-Prior L. (2018). Objective measures of gait and balance in healthy non-falling adults as a function of age. Gait Posture.

[39] Coni A., Mellone S., Colpo M., Guralnik J.M., Patel K.V., Bandinelli S., Chiari L. (2019). An Exploratory Factor Analysis of Sensor-Based Physical Capability Assessment. Sensors.

[40] Tang W., Fulk G., Zeigler S., Zhang T., Sazonov E. Estimating Berg Balance Scale and Mini Balance Evaluation System Test Scores by Using Wearable Shoe Sensors. Proceedings of the 2019 IEEE EMBS International Conference on Biomedical & Health Informatics (BHI).

[41] Bagala F., Becker C., Cappello A., Chiari L., Aminian K., Hausdorff J.M., Zijlstra W., Klenk J. (2012). Evaluation of Accelerometer-Based Fall Detection Algorithms on Real-World Falls. PLoS ONE.

[42] Alex M., Chen C., Wünsche B.C. A review of sensor devices in stroke rehabilitation. Proceedings of the 2017 International Conference on Image and Vision Computing New Zealand (IVCNZ).

[43] Shany T., Wang K., Liu Y., Lovell N.H., Redmond S.J. (2015). Review: Are we stumbling in our quest to find the best predictor? Over-optimism in sensor-based models for predicting falls in older adults. Healthc. Technol. Lett..

[44] Isaksson A., Wallman M., Göransson H., Gustafsson M.G. (2008). Cross-validation and bootstrapping are unreliable in small sample classification. Pattern Recognit. Lett..

[45] Donoghue D., Stokes E.K. (2009). How much change is true change? The minimum detectable change of the Berg Balance Scale in elderly people. J. Rehabil. Med..

[46] Dunne L., Profita H., Zeagler C. (2014). Social aspects of wearability and interaction. Wearable Sensors.

